# Strategies to Enrich Electrochemical Sensing Data with Analytical Relevance for Machine Learning Applications: A Focused Review

**DOI:** 10.3390/s24123855

**Published:** 2024-06-14

**Authors:** Mijeong Kang, Donghyeon Kim, Jihee Kim, Nakyung Kim, Seunghun Lee

**Affiliations:** 1 Department of Optics and Mechatronics Engineering, College of Nanoscience & Nanotechnology, Pusan National University, Busan 46241, Republic of Korea; 2 Department of Cogno-Mechatronics Engineering, College of Nanoscience & Nanotechnology, Pusan National University, Busan 46241, Republic of Korea; 3 Department of Physics, Pukyong National University, Busan 48513, Republic of Korea

**Keywords:** electrochemical sensor, voltammetry, machine learning, identification, quantification

## Abstract

In this review, recent advances regarding the integration of machine learning into electrochemical analysis are overviewed, focusing on the strategies to increase the analytical context of electrochemical data for enhanced machine learning applications. While information-rich electrochemical data offer great potential for machine learning applications, limitations arise when sensors struggle to identify or quantitatively detect target substances in a complex matrix of non-target substances. Advanced machine learning techniques are crucial, but equally important is the development of methods to ensure that electrochemical systems can generate data with reasonable variations across different targets or the different concentrations of a single target. We discuss five strategies developed for building such electrochemical systems, employed in the steps of preparing sensing electrodes, recording signals, and analyzing data. In addition, we explore approaches for acquiring and augmenting the datasets used to train and validate machine learning models. Through these insights, we aim to inspire researchers to fully leverage the potential of machine learning in electroanalytical science.

## 1. Introduction

**Figure 1:**
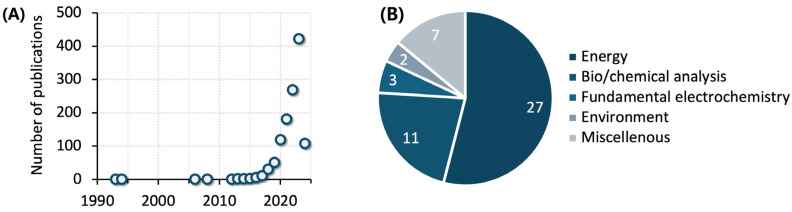
(**A**) The number of articles with ‘electrochemistry’ and ‘machine learning’ as their topic published up to the present (the first quarter of 2024). (**B**) The number of review articles with ‘electrochemistry’ and ‘machine learning’ as their topic, published from 2021 to the present. The review articles are categorized based on the research field.

Since the early 2010s, the integration of machine learning into electrochemical research has been embarked on in a wide range of fields such as energy generation/storage [1,2,3,4], bio/chemical analysis [5,6,7], fundamental electrochemistry [8,9], and environmental sustainability [10] (Figure 1A). The main goals of machine learning applications are, from a material point of view, to improve the functionality of materials utilized in electrochemical research and, from an analysis point of view, to facilitate data processing and interpretation [11]. As evidenced by the recently published review articles pertaining to both electrochemistry and machine learning (Figure 1B), the energy-related research field has most extensively embraced machine learning for synthesizing, optimizing, and evaluating materials and devices. In the field of bio/chemical analysis, machine learning is employed to enhance information extraction from electrochemical data, which has been a major challenge, especially when analyzing real-world samples with complex compositions. The qualitative and/or quantitative analysis of such complex samples often necessitates a series of functionalizations of sensing electrode surfaces—a resource- and time-intensive step that could be less productive than expected. Machine learning can uncover hidden information from the noisy data obtained at a single electrode or few electrodes and help mitigate the costs associated with the fabrication of electrochemical sensors while enhancing the efficiency of the analysis.

**Figure 2:**
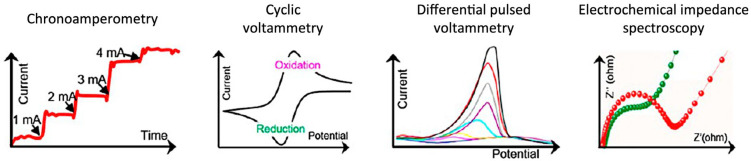
Electrochemical measurement techniques and typical data plots. Reproduced with permission from ref. [12]. Copyright 2021, American Chemical Society (Washington, DC, USA).

In electrochemical analysis, a variety of measurement techniques are used, and unique data are generated with different dimensionalities. Figure 2 shows some of the commonly used electrochemical techniques and their typical data plots [12]. Chronoamperometry (CA) measures currents continuously while a constant potential is applied to the sensing electrode, yielding a one-dimensional current vs. time data. As the electrolyte solution is exposed to a target substance, the current level can immediately increase, with the increment often proportional to the target concentration. In cyclic voltammetry (CV), a potential applied to the sensing electrode is linearly scanned in a positive or negative direction and then reversed (to a negative or positive direction), and currents are continuously recorded vs. their potential. Both oxidation and reduction currents can be measured symmetrically, as shown in Figure 2, or only one of them can be observed depending on the nature of the electrochemically active substance. In differential pulsed voltammetry (DPV), the potential pulses are added to a linear potential scan, currents are sampled twice, right before the onset and the end of each pulse, and the difference in these two current measurements is plotted vs. potential. In general, such a differential technique enhances the sensitivity and selectivity of measurements. Beyond these one-dimensional data, a higher-dimensional one is also obtainable. In electrochemical impedance spectroscopy (EIS), the current in response to an oscillating potential is measured at several oscillation frequencies (usually tens of frequencies within the range from 1 mHz to 1 MHz), and the real and imaginary components of electrochemical impedance are plotted, as illustrated in Figure 2 [13]. This high-dimensional EIS plot is usually interpreted with equivalent circuit models [14]. To extract information from electrochemical data, various machine learning models (e.g., decision tree, support vector, artificial neural network) are used. For details on machine learning techniques, the readers may refer to other literature [11,15,16].

Although there is plenty of room in electrochemical data for machine learning applications, the application can be limited if the performance of electrochemical sensors is not good enough to sense a target in a real-world sample crowded with numerous electroactive non-target substances. Therefore, together with advanced machine learning techniques, a method to enhance the relevance of electrochemical sensing data to target substances needs to be developed to maximize the performance of machine-learning-assisted electrochemical analysis. In this review, we introduce five recently reported strategies employed to render any difference in electrochemical data for different analytes or the different concentrations of a single analyte. Then, we explain how the information-bearing features of electrochemical data are defined. Since it is one of the major concerns of electrochemical analysts to obtain a large amount of data from a single electrode or a small array of electrodes, we present the quantity of data and/or the augmentation of data used for training and validating machine learning models. We hope that this review can provide the readers with some ideas to maximize the potential of machine learning in electroanalytical science.

## 2. Strategy I. Composing a Set of Electrodes That Differently Interact with Target Substances to Enrich the Diversity of the Electrochemical Dataset

Antibiotics protect food-producing livestock against diseases caused by bacterial species, but their residual presence in food exposes humans to health risks, such as direct disturbances to human physiology [17]. This leads to the development of sensors that are capable of detecting and identifying antibiotics in food samples. Traditional analytical methods typically involve separation (e.g., using chromatography) and detection (e.g., using mass spectrometry) steps, which are both highly resource- and time-intensive. In this context, simple and rapid electrochemical measurements have been developed for detecting antibiotics, but they often suffer from a limited capacity to unambiguously categorize signals from multiple antibiotic molecules in a complex sample [18,19].

**Figure 3:**
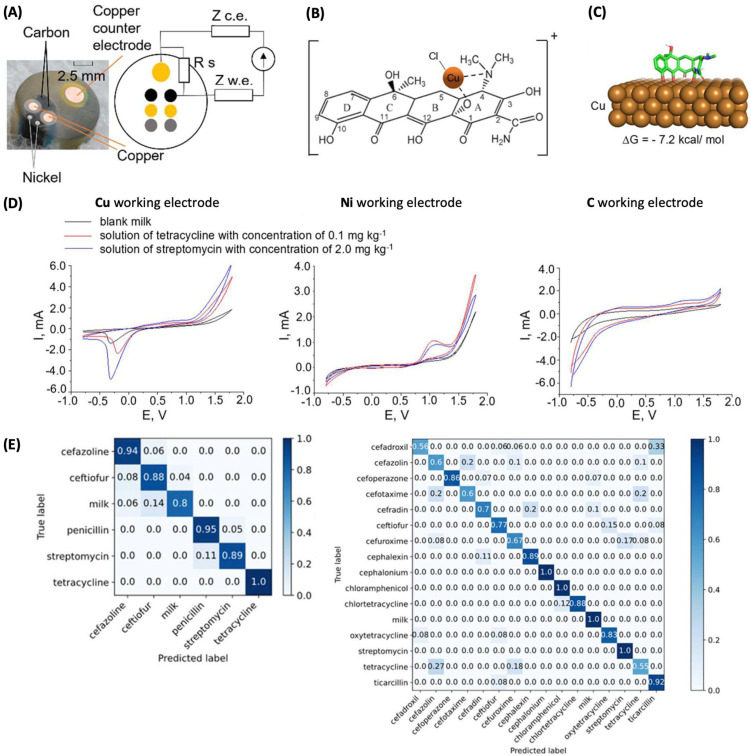
(**A**) A multi-electrode sensor composed of Cu, Ni, and C working electrodes and a Cu counter electrode. (**B**) The proposed coordination interaction of tetracycline with the Cu^+^ of CuCl. (**C**) The predicted adsorption of tetracycline on the Cu surface. (**D**) Cyclic voltammograms obtained from milk solutions in the presence and absence of antibiotic molecules, measured by Cu, Ni, and C working electrodes. (**E**) Confusion matrices indicating the classification accuracy for 5 and 15 antibiotic molecules. Reproduced with permission from ref. [20]. Copyright 2023, American Chemical Society (Washington D.C., USA).

To improve the identification of multiple antibiotics in milk, T. A. Aliev et al. used a multi-electrode system composed of Cu, Ni, and C working electrodes (sharing a Cu counter electrode, Figure 3A) that differently responded to the antibiotic molecules and generated a complementary dataset [20]. Metal elements have their own oxidation and reduction characteristics and result in unique cyclic voltammograms. For a single metal element, multiple redox reactions can take place, where both metal cations (Cu^2+^, Cu^+^, or Ni^2+^) and/or metal-containing species (e.g., for copper, Cu_2_O, Cu(OH)_2_, or CuCl; for nickel, NiOOH, NiO_2_, NI(OH)_2_) are involved. Their chemical state affects the reversibility of their redox reaction, and any alteration in the chemical state (e.g., activity or concentration) can subtly modify the shape of the CV curve. Meanwhile, an individual antibiotic can interact with the metal cation via coordination bonding (Figure 3B) and with the metal surface via its adsorption (Figure 3C). Such interactions influence the redox reaction of the metal, which is significantly dependent on the metal element. For instance, the current peak around −0.3 V and the current plateau from +0.3 to +1.2 V in the CV curve obtained by a Cu working electrode (black curve in Figure 3D), which is attributed to the oxidation reactions at the Cu working electrode and Cu counter electrode, respectively. One of the Cu-containing species that can be formed in milk is CuCl, and it can be electrochemically reduced to generate Cu^0^, which can participate in the electrochemical oxidation of Cu electrodes. When the milk sample contains tetracycline, an antibiotic, it reacts with CuCl to form a complex compound, as illustrated in Figure 3B. This alters the relative concentrations of Cu-containing species involved in Cu oxidation and can contribute to the shift in the oxidation peak potential (red curve in Figure 3D). In addition, tetracycline can be adsorbed on the Cu surface, as modeled in Figure 3C, which can affect the electrochemical process. In this way, the three different electrodes yield a unique set of sensing signals for individual antibiotics, and the signals can be combined to yield an electrochemical fingerprint of an antibiotic molecule.

To identify the antibiotic molecules present in milk from the cyclic voltammogram, the authors used machine learning equipped with several algorithms including decision trees and random forests. Before feeding the model with data, they first converted a CV curve (i.e., the current as a function of the potential scanned bidirectionally) to a current–time curve (i.e., a unidirectional reading of currents over time). A single (converted) cyclic voltammogram consisted of 1040 current values, and all these values were taken as features.

To train and validate the machine learning model, milk samples containing a single antibiotic molecule were prepared at five different concentrations, with a total number of 15 antibiotics investigated. When obtaining the electrochemical dataset from this large number of samples, the multi-electrode system was reused, with the electrode surfaces mechanically polished after every three cycles. For training and validating the model to discriminate five antibiotics, in other words, to classify data into six classes (one negative control (i.e., milk without antibiotics added) and the five antibiotics), a total of 1377 cyclic voltammograms were used. The dataset was divided into the training and validating sets at ratios of 1:1, 8:2, and 9:1, and the ratio that yielded the best model was selected. The model achieved classification accuracies ranging from 0.8 to 1.0, as shown in the left confusion matrix in Figure 3E. (Note: the confusion matrices in Figure 3E indicate how correctly the trained model operates. Higher values in the diagonal of a matrix indicate more accurate classification.) For the second dataset consisting of 2122 cyclic voltammograms from 16 classes (one negative control and fifteen antibiotics), although more data were available, the amount of data for each class was lower. Consequently, a comparatively lower accuracy of classification (ranging from 0.55 to 1.0) was obtained, as shown in the right confusion matrix in Figure 3E. This result underscores the importance of having sufficient data for each class to achieve accurate predictions by machine learning.

As a key factor in Strategy I, a combination of electrodes with different responsiveness to a detection target can be prepared using the same electrode materials with different states of modification. T. Shingu et al. electrochemically oxidized carbon nanotube (CNT) electrodes at different potentials, altering the crystallinity (i.e., by creating defects) and chemical properties (i.e., by forming functional groups) of CNT [21]. This modification introduced nonlinearity to the electrode properties (e.g., electric double-layer capacitance, electron transfer rate), resulting in diverse electrochemical sensing signals. Using four differently oxidized CNTs, the authors fabricated multi-electrode sensing devices and employed them to predict the blood glucose level with the aid of a neural network algorithm: reservoir computing. As exemplified by this study, various approaches to fabricating multi-electrode systems expand the potential for machine-learning-powered electrochemical sensing.

## 3. Strategy II. Leveraging the Versatility of Electrochemical Measurements to Generate Bigger Data for Individual Target Substances

**Figure 4:**
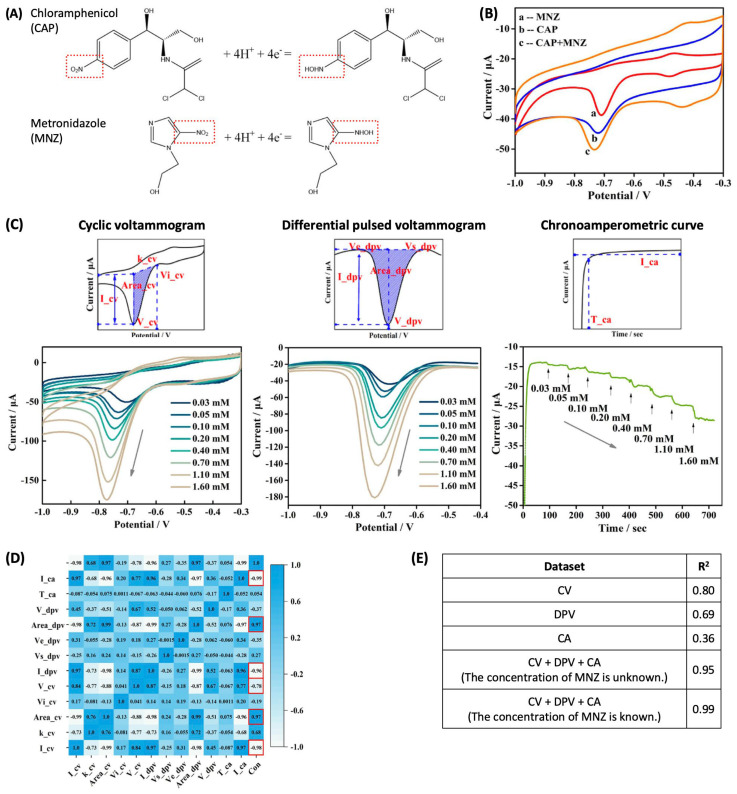
(**A**) Four-electron reduction reactions of chloroamphenicol (CAP) and metronidazole (MNZ). (**B**) Cyclic voltammograms of MNZ, CAP, and their mixture. (**C**) Features of cyclic voltammogram, differential pulsed voltammogram, chronoamperometric curve, and the electrochemical data with the increasing concentration of CAP. (**D**) Pearson correlation between features and CAP concentrations. (**E**) R^2^ (indicating the goodness-of-fit) of a linear fit applied to the plot of true concentration vs. predicted concentration, calculated from different datasets. Reproduced with permission from ref. [22]. Copyright 2023, Institute of Electrical and Electronics Engineers Inc (Piscataway, NJ, USA).

The antibiotic molecules possessing their electrochemical reactivities can directly generate detection signals. In this case, there is a major challenge in distinguishing between those with similar redox-active moieties. For instance, chloramphenicol (CAP) and metronidazole (MNZ) both have a nitro group (-NO_2_) and undergo a four-electron reduction (Figure 4A) in a comparable potential range (Figure 4B, reduction peaks of both antibiotics appear around −0.7 V). This indicates that the selective detection of either one is hindered by the other. This challenge can be addressed by leveraging the great versatility of an electrochemical measurement. CV, DPV, and CA examine an analyte from different aspects and yield distinct output data. A series of these measurement techniques can enrich the dataset with more information on the analytes. Such a high-dimensional dataset is well-suited for machine learning; however, a challenge that arises from this aspect is the increased risk of overfitting. (Note: overfitting occurs when the machine learning model fits too closely to the training data, possibly resulting in poor generalization (i.e., the model does not give accurate predictions for new data)). This is commonly seen when the input data are of a high dimension while the number of training data is relatively low.

Y. Xu et al. applied machine learning to high-dimensional data composed of CV, DPV, and CA curves for the detection of CAP in the presence of MNZ and, in particular, reduced the dimension of data in two steps to avoid overfitting [22]. From a cyclic voltammogram, several features of the reduction peak can be defined, as shown in Figure 4C. For the right-side half of the reduction peak, first, a linear baseline can be obtained by connecting the two endpoint currents. Using the peak and the baseline, a peak current (I_cv), peak potential (V_cv), onset potential (Vi_cv), the area enclosed by the peak (Area_cv), and the slope of the baseline (k_cv) can be defined. Different sets of features in the DPV and CA curves are also obtainable, as described in Figure 4C. These parameters reduce a set of three one-dimensional raw data to a parametric matrix, but it is still of high dimension. To further reduce the dimensionality, Pearson’s correlation was used to identify the parameters highly relevant to the sought information (i.e., CAP concentration): three parameters (I_cv, Area_cv, and V_cv) in a cyclic voltammogram, two (I_dpv, Area_dpv) in a differential pulsed voltammogram, and one (I_ca) in a chronoamperometric curve. Figure 4D shows the strength of the correlation between features and the concentrations of CAP. The selected features were then input into a feedforward backpropagation artificial neural network (ANN). When a set of features from a single measurement was used for modeling, the ANN model yielded the prediction accuracies of 0.80, 0.69, and 0.36 for CV, DPV, and CA datasets, respectively (Figure 4E). (Note: R^2^ in Figure 4E indicates the goodness-of-fit of a linear fit applied to the plot of true concentration vs. predicted concentration. Higher values indicate more accurate quantification.) When a set of assorted features from all three measurements was used, the prediction performance was improved significantly, with a prediction accuracy of 0.95. This unambiguously indicates that the combination of multiple electrochemical measurement techniques enhances the analytical performance of machine learning.

To obtain the dataset, CAP solutions were prepared at 8 different concentrations in the presence of MNZ at 3 different concentrations, and each solution was investigated 20 times in duplicate using CV, DPV, and CA. A total of 1440 datasets were obtained and randomly divided into the training and validating sets at a ratio of 4:1.

To fully leverage Strategy II, it is critical to choose the best combination of the feature-extracting methods and training algorithms for specific data types. D. K. X. Teo et al. demonstrated that predicting acetaminophen levels from DPV curves and carcinoembryonic antigen levels from EIS spectra varied significantly based on the feature extraction methods (principal component analysis, linear discriminative analysis, fast Fourier transfer, discrete wavelet transform) and training algorithms (linear regression, support vector regression, multilayer perceptron) used [23]. Based on performance metrics (coefficient of determination, root mean square error, mean absolute error, average relative error, F1 score), the authors found that multilayer perceptron with discrete wavelet transform was best for the DPV dataset and multilayer perceptron with principal component analysis was best for the EIS dataset. This study underscores the importance of optimizing both feature extraction and machine learning tools.

## 4. Strategy III. Dissecting an Electrochemical Signal to Catch the Trace-Level Quantity of a Target Substance in a Complex Medium

In a biological system, there are certain substances that play essential roles in maintaining physiological processes but which can also contribute to pathogenetic processes when present at abnormal concentrations. Therefore, the concentrations of such materials are homeostatically maintained in a living system. For instance, certain transition metal cations (e.g., Cu^2+^) at trace levels are essential for cell physiology (e.g., serving as cofactors for enzymes associated with energy generation) [24], but at abnormal concentrations can lead to cell death and various diseases (e.g., cardiovascular diseases, neurodegenerative diseases, cancers) [25]. In research involving cell culture (e.g., the preparation of organ-on-chip models), the concentration of a transition metal cation in a cell culture medium is of great interest [26]. For this, electrochemical measurements can offer direct, non-destructive, and real-time monitoring since many biologically relevant transition metal cations can be electrochemically reduced at their own reduction potentials. For instance, the standard reduction potential of Cu^2^ (for the reaction Cu^2+^ + e^−^ → Cu^+^) is +0.15 V vs. the standard hydrogen electrode, and Cu^2+^ in a cell culture medium can be deposited on an electrode immersed in the media and polarized at a potential lower than +0.15 V. The electrodeposited Cu film can then be oxidatively stripped as the potential is scanned from a level more negative than +0.15 V toward the positive direction, resulting in the well-defined peak of the oxidation current; this process is termed anodic stripping voltammetry (ASV). By analyzing the oxidation peak in the ASV curve, it is possible to identify and quantify the transition metal cation in a cell culture medium. The culture medium, however, contains many other substances (e.g., glucose, amino acids, minerals, vitamins, antibiotics) that can affect the electrochemical reactions, making the selective detection of a transition metal cation challenging.

**Figure 5:**
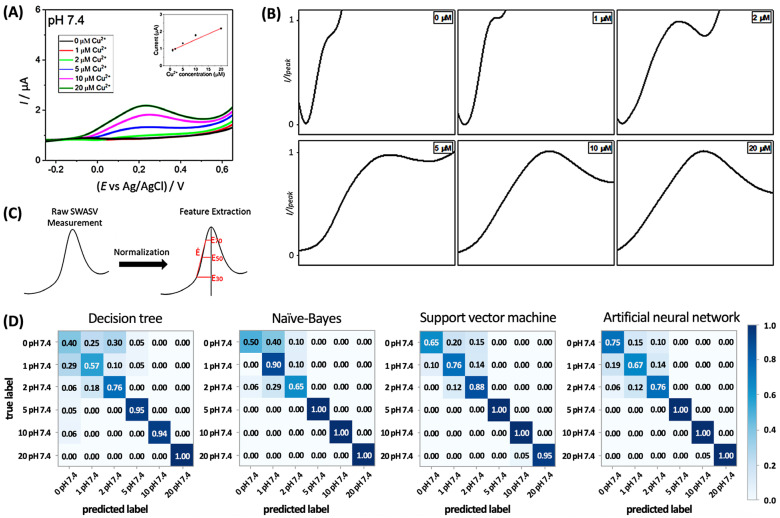
(**A**) Anodic stripping voltammetry (ASV) curves for cell culture media with different concentrations of Cu^2+^ dissolved. (**B**) Normalized ASV curves. (**C**) Features of the oxidation peak in the normalized ASV curve. (**D**) Confusion matrices indicate the performances of different machine learning models for predicting Cu^2+^ concentrations. Reproduced with permission from ref. [26]. Copyright 2023, MDPI (Basel, Switzerland).

For the electrochemical monitoring of the concentration of the biologically significant Cu^2+^ in cell culture media, F. Biscalia et al. utilized a modified ASV technique (that is, square wave ASV), sophisticatedly analyzed the shape of an ASV curve, and found out the Cu^2+^ concentration via machine learning [26]. Figure 5A shows the ASV curves obtained from commonly used cell culture media (that is, the minimum essential Eagle’s medium) containing different concentrations of Cu^2+^. The ASV curve gradually transforms with the increasing Cu^2+^ concentration. In a typical ASV-based analysis, the magnitude of the oxidation peak current or the charge associated with the oxidation peak is used for quantifying the metal cation [27]. In contrast, F. Biscalia et al. normalized the ASV curve (Figure 5B) and defined a set of features for a single current peak, as described in Figure 5C. For feature extraction, only half of the peak at a lower potential side was used as the current at a higher potential is more susceptible to other processes (e.g., oxidation of water) than Cu stripping. The potential-related features were defined as the half widths at 30, 50, and 70% of the maximum current, denoted as E_0.3_, E_0.5_, and E_0.7_, respectively. For these features, the ratio E_0.3_/E_0.7_ and the derivative in the ascending part can offer a detailed description of the shape of an oxidation peak. For each dataset, a set of features and the corresponding class (i.e., Cu^2+^ concentration) were input into different machine learning models, which included decision trees, naïve-Bayes, support vector machine, and artificial neural networks, and their performances were compared (Figure 5D). Support vector machine was found to be the most accurate in this application.

To successfully apply a machine learning model, a large dataset is required during the training of the model. For this, the authors implemented data augmentation using the random perturbation method. This method synthesizes new ASV curves from an experimentally obtained curve by randomly perturbing it with the standard deviations of several experimentally obtained curves in the same class. This augmentation effectively increased the size of the dataset by the eighth fold and helped mitigate the risk of overfitting.

For Strategy III to be successful in sensing a target substance present at extremely low concentrations, information-denser electrochemical signals with sophisticated feature extraction from these signals may be necessary. Y. Zhao et al. fabricated an electrochemical sensor for detecting insulin (physiological level: 35 to 145 pM) and glucose (physiological level: 3.9 to 6.1 mM), the simultaneous detection of which is beneficial for diabetes diagnosis [28]. Given the significantly low concentration of insulin (over 10^7^ times lower than that of glucose), a highly sensitive method is needed to capture the subtle response of the sensing electrode to insulin. The authors prepared a Ni-based electrode with inherent redox activity, which was perturbed by insulin and glucose to different extents (this type of approach is introduced in Strategy IV). Cyclic voltammograms were obtained to observe the redox activity of the electrode in the presence of insulin and glucose at various concentrations, and the shape of the CV curves was scrutinized to yield seven features. These features were fed into a linear regression algorithm, enabling the prediction of insulin concentration from 1 to 250 pM for the CV curves. Furthermore, the model predicted the concentration of insulin in highly crowded clinical serum samples with less than 20% error, meeting accuracy criteria based on the ISO standard.

## 5. Strategy IV. Adopting an Electrochemical System That Is Perturbed in Different Ways by Different Target Substances and Identifying Targets from the Perturbation

Numerous hazardous gases (e.g., volatile organic compounds (VOCs) such as aldehyde, methanol, and acetone) are frequently found in various commercial products and indoor/outdoor environments [29]. Since many gaseous molecules have electrochemical reactivities at mild electrode potential, electrochemical sensors have been used to monitor the environmental risks caused by such molecules. In many cases, chronoamperometry is utilized as a real-time monitoring technique [30]. Although individual molecules have their own reactivity, their electrochemical responses, at least in part overlap, and the resultant cross-sensitivity is a common challenge of the electrochemical gas sensors. One approach to improve the sensing selectivity is to modify the electrode surface with functional materials that have different levels of responsiveness to different gas molecules. For instance, an enzyme (e.g., aldehyde dehydrogenase [31]), which has a high specificity to its substrate (e.g., formaldehyde), can be tethered to the electrode surface to detect the single target substrate. However, for multiplex gas detection, this approach requires multiple recognition components and an array of individual electrodes for each recognition component, which inevitably increases the cost of detection.

**Figure 6:**
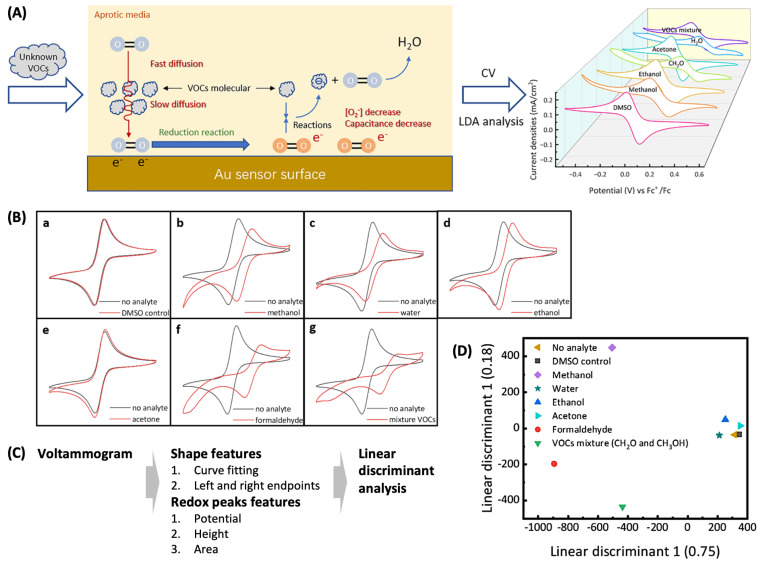
(**A**) A rationally designed electrochemical system consisting of an O_2_/O_2_^−^ redox couple and an ionic liquid that responds to volatile organic compounds (VOCs) and the variable cyclic voltammograms in the presence of various VOCs. (**B**) Characteristic cyclic voltammograms for O_2_/O_2_^−^ obtained in dimethyl sulfoxide in the absence (black curves) and presence (red curves) of specific VOCs. (**C**) Data analysis flowchart. (**D**) Linear discriminant analysis diagram. Reproduced with permission from ref. [32]. Copyright 2023, American Chemical Society (Washington D.C., USA).

For multiplex gas sensing using a single sensing electrode, X. Huan et al. utilized a well-established redox reaction that was affected differently by different VOCs and employed machine learning to identify VOCs by recognizing the little-to-substantial variation in electrochemical signals [32]. The authors observed a reversible reaction of a O_2_/O_2_^−^ redox couple in a solution containing dimethyl sulfoxide (DMSO) as a solvent and an ionic liquid, 1-butyl-1-methylpyrrolidium bis(trifluoromethylsulfonyl)imide that could physically or chemically interact with VOCs (Figure 6A). When this redox reaction was investigated with CV in the presence of various VOCs (methanol, ethanol, acetone, formaldehyde, and VOCs mixture) and water, the CV curves showed different responses to each VOC (Figure 6B). For the reversible O_2_/O_2_^−^ reaction to yield a symmetric CV curve (black curves in Figure 6B), the O_2_ transported from the bulk solution to the electrode surface via diffusion should be reduced in the cathodic scan, and the resultant O_2_^−^ should be oxidized in the following anodic scan. VOCs obtain different chemical properties and alter the CV curves in three different ways. First, the VOCs that have relatively labile H^+^ (shortened as AH: methanol and ethanol fall in this category) can interact with O_2_^−^ to form products (AH^−^ and O_2_) reversibly, and then these products can irreversibly form H_2_O and A^−^. These chemical reactions influence the concentration of O_2_ and O_2_^−^ near the electrode surface, and the characteristics of a cyclic voltammogram are altered consequently. The extent of the alteration increases (methanol > water > ethanol) with increasing the constant rate for the reaction between O_2_^−^ and AH^−^ (note: the rate constants for methanol, water, and ethanol are in the orders of 10^7^, 10^5^, and 10^2^, respectively). Another VOC, aldehyde, reacts with O_2_^−^ to form H_2_O and CO_2_, and these products consume O_2_^−^, resulting in a dramatic alteration of the cyclic voltammogram. Finally, the VOC with negligible reactivity to O_2_^−^, which is acetone, does not induce a significant shape alteration of a cyclic voltammogram but small increases in the oxidation and reduction currents due to the higher diffusion coefficient of O_2_^−^ in acetone than in DMSO. In summary, the individual VOCs with unique chemical natures interact with O_2_^−^ in specific ways and result in the CV curves of unique characteristics (“fingerprint”). The authors extracted several features from the CV curves to discriminate VOCs using machine learning (see details below). This approach does not require a set of individual sensors for detecting specific VOCs; instead, a single electrochemical system rationally designed to have semi-specific responses to various VOCs is used with the aid of machine learning for VOC discrimination.

To classify cyclic voltammograms in Figure 6B into the correct VOC classes, linear discriminant analysis (LDA) was adopted as a machine learning algorithm (Figure 6C). Voltammograms can serve as information-rich source data for machine learning [33]; this has other aspects, such as the necessity of a greater amount of resources for processing. To avoid this pitfall, the raw data matrix can be projected to a lower dimensional space. In this study, a cyclic voltammogram was divided into eight segments and then fitted with a cubic polynomial (I = aV^3^ + bV^2^ + cV + d, where I is the current and V is the potential). The fitting parameters a, b, c, and d and the current values at the left and right ends of each segment were used as the features of an individual cycle of a CV curve. In addition, the characteristics of the redox peak, which are the height, area, potential of a redox peak, and the difference between oxidation and reduction peak potentials, were also used to describe the shape of the CV curve. LDA first extracted these features to reduce the dimensionality of cyclic voltammograms. Next, using the class label (i.e., the identity of a VOC) given for each cyclic voltammogram, LDA combined the features to maximize class separability. This involved weighing the features to increase interclass variance and decrease intraclass variance, resulting in a combination of features termed the discriminant function. Figure 6D shows the LDA diagram of two linear discriminants that most significantly discriminated different classes. Each VOC had its own position in the diagram, indicating how LDA effectively discriminated against VOCs.

To train the LDA to discriminate VOCs, a single cyclic voltammogram was obtained with ten potential cycles. The first cycle was used as a stabilizing process and was not used for analysis, and the nine cyclic voltammograms for each VOC were used for training. (Note: the process for validating the machine learning model using a different dataset was not conducted in this study.)

Another example that demonstrates the use of Strategy IV involves the redox reaction of a transition metal cation within a complex ion, wherein the redox behavior is dependent on the chemical properties of ligands [34]. S. Deb et al. synthesized a Ru(II) complex ion containing a deprotonatable ligand, of which deprotonation decreased the net positive charge in the complex ion, resulting in the negative shift of the potential for a Ru(II)/Ru(III) reaction. By observing that more deprotonation was induced by anions with stronger basicity, the authors exploited this relationship to sense anions. Voltammograms (along with spectroscopic data) of Ru(II) complex ions in the presence of different anions were used to predict the sensing characteristic of the complex ion via fuzzy logic, ANN, or the adaptive neuro-fuzzy interference system. This example, along with the study shown in Figure 6, illustrates that the wide range of redox systems, when combined with machine-learning-based analysis, can be used to indirectly sense a target substance.

## 6. Strategy V. Using Raw Voltammetric Data without Post-Processing to Reduce the Processing Cost for Machine Learning in Point-of-Care Applications

**Figure 7:**
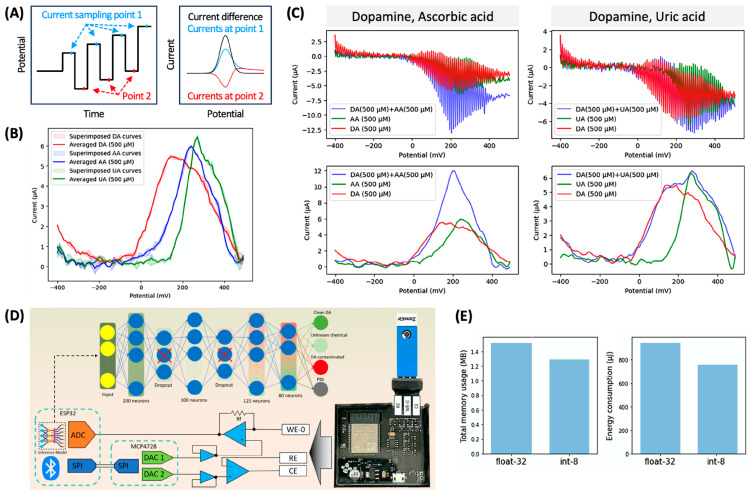
(**A**) Input potential and output current in square wave voltammetry (SWV). (**B**) Typical SWV curves for individual dopamine (DA), ascorbic acid (AA), and uric acid (UA). (**C**) Unprocessed (upper) and typically processed (lower) SWV curves for DA, interfering species (either AA or UA), and their mixture. (**D**) Electrochemical measurement and data analysis flowchart. (**E**) Resources (total memory usage and energy consumption) used for machine learning of the unprocessed data with 32-bit float point units (float-32) or after 8-bit quantization (int-8). Reproduced with permission from ref. [37]. Copyright 2023, Wiley-VCH GmbH (Hoboken, Germany).

Electrochemical measurements offer a significant advantage in point-of-care (POC) applications due to their portability, exemplified by handheld blood glucose meters. There is a growing interest in developing electrochemical POC sensors to monitor the levels of clinically significant biomolecules even at the bedside [35]. Many biomolecules exhibit reactivities for electrochemical oxidation and reduction within a mild potential range. In other words, achieving high specificity for a target biomolecule over interfering species is a great challenge in electrochemical biosensors. One solution from a signal-recording perspective is to adopt an advanced differential technique, such as square wave voltammetry (SWV), which combines the advantages of CV, DPV, and EIS [36]. As illustrated in Figure 7A, SWV applies both anodic and cathodic potential pulses, samples currents right before the end of each pulse, and finally displays the difference in currents sampled at two sets of points. The relatively complicated signal processing in SWV, however, imposes some processing penalties, which is critical for low-powered processors used in portable POC sensors. Furthermore, even with SWV, it is challenging to separate signals from certain biomolecules, such as dopamine, ascorbic acid, and uric acid (Figure 7B).

For the POC detection of dopamine (DA), an essential neurotransmitter, in the presence of two common interfering species, ascorbic acid (AA) and uric acid (UA), J. I. O. Filho et al. developed a SWV-based sensor equipped with the machine learning processor operating with unprocessed SWV data for reducing the burden of processing [37]. Figure 7C shows the unprocessed data (i.e., raw current data in response to the square-wave potentials) as well as typical SWV curves obtained via signal processing in general electrochemical instruments. The processed SWV curves show that the individual oxidations of dopamine, ascorbic acid, and uric acid share the potential range in part. Therefore, to extract useful information (in this study, whether dopamine is present in the sample and whether interfering species are present as well) from the data obtained from mixtures (DA + AA, DA + UA, and DA + AA + UA), additional processing was required. In contrast, the raw SWV data consisting of 182 data points in 32-bit float point unit (float-32) were obtained by the handheld instrument developed in this study and were input into the machine learning processor directly or after 8-bit quantization (int-8) (Figure 7D). The neural network-based machine learning model classified the data into Con DA (contaminated DA, representing that the solution contains contaminants), Cle DA (clean DA, representing that the solution contains only DA), PBS (phosphate-buffered saline, representing a blank solution), and UC (unknown chemical, representing that the solution has no DA but other chemicals) at accuracies over 95%. More importantly, this process occupied ≈15.67% and ≈4.55% of the total memory when run with float-32 and int-8 data, respectively, and consumed the energy at sub-mJ levels. (Figure 7E). (Note: for a commercial electrochemical instrument ADuCM355, only the model with quantized data fitted and occupied 72.89% of the flash memory for the classification process.) The model can be improved simply by having the trade-off between memory and accuracy. The machine learning model used in this study without data pre-processing is well-suited for low-powered processors, which can accelerate the integration of machine learning to electrochemical POC sensors.

For data collection, solutions with 55 different concentrations of DA, AA, and UA were prepared, and each solution was investigated in triplicate, resulting in 165 SWV curves. SWV data were augmented by jittering, scaling, jittering with scaling, and random scaling at random points, generating 25 SWV curves for each concentration of DA, AA, UA, their mixtures, and a blank buffer. With some additional measurements, a total of 5492 data were obtained and divided at a ratio of 4:1 for training and validating the machine learning model.

## 7. Conclusions

Machine learning has helped researchers in the field of analytical science to extract useful information from limited datasets beyond what was previously expected. Electrochemical measurements, in particular, have been considered hardly suitable for generating a massive dataset. However, recent advancements in multi-electrode systems (e.g., screen-printed electrode array integrated with a 96-well plate commonly used in spectroscopic measurements) [38] have enhanced this capability. As these developments continue, machine learning is expected to play a much larger role in electrochemical analysis. Many efforts have been made to develop advanced machine learning models with new algorithms and techniques. However, it should be noted that the output of machine learning is highly dependent on the quality of experimental data. Machine learning can provide valuable output when there is at least a subtle correlation between the experimental data and the information sought, such as the identity and/or concentration of target species. This review outlines recently developed strategies to enrich electrochemical data with relevance to the sought information. Strategies I and II focus on increasing the number of information-bearing features during sensing preparation and signal recording, respectively. Strategies III and IV are implemented in the signal analysis stage to capture information-correlated subtleties of the features.

Furthermore, a strategy for the practical operation of electrochemical sensors is introduced (Strategy V). While electrochemical sensors have great potential in POC applications, they generally suffer from relatively poor performance. This is an area where machine learning can offer substantial benefits, and we expect that there will be more integrations of machine learning into electrochemical POC sensors, which are currently undergoing rapid development. In this context, finding methods to reduce the resource consumption of a sensing device can lead to a great leap forward for electrochemical POC sensors [39].

Alongside the improvement of machine learning methods, even small but creative modifications to electrochemical sensing systems can result in tremendous advancements in analytical science. Machine learning can be directly used for such modifications. Recent advances in autonomous systems employing high-throughput experimentation and machine learning-assisted real-time data analysis further accelerate this process, enabling rapid and iterative sensor design optimization [40].
